# Comprehensive assessment of ground motion amplification in stratified soils with different layer configurations and types

**DOI:** 10.1038/s41598-026-35581-8

**Published:** 2026-01-14

**Authors:** Asadullah Ziar, Ender Basari

**Affiliations:** 1 Department of Civil Engineering, Ghazni Technical University, Ghazni, 2301 Afghanistan; 2 Department of Civil Engineering, Manisa Celal Bayar University, Manisa, 45140 Turkey

**Keywords:** Seismic site response, Stratified soils, Spectral acceleration, Ground motion amplification, And rsseismic, Engineering, Environmental sciences, Natural hazards, Solid Earth sciences

## Abstract

This study investigates the seismic response of thirty meter deep soil profiles with varying compositions and layering sequences, including homogeneous clay and sand profiles and partially layered profiles composed of 22.5 m of clay over 7.5 m of sand, 7.5 m of clay over 22.5 m of sand, 22.5 m of sand over 7.5 m of clay, 7.5 m of sand over 22.5 m of clay, and evenly layered profiles with 15 m of clay over 15 m of sand or 15 m of sand over 15 m of clay. Nonlinear one-dimensional ground response analyses were performed using RSSeismic software, applying seven strong ground motions scaled to peak ground acceleration levels of 0.10 g, 0.25 g, and 0.50 g. The results demonstrate that seismic amplification is strongly governed by the soil type located at the ground surface, impedance contrasts between adjacent layers, thickness distribution of soft and stiff materials, and nonlinear stiffness degradation under increasing shaking intensity. Profiles with clay at the surface consistently produce higher amplification and longer period response because of greater modulus degradation, whereas sand dominated surfaces generate stronger short period amplification with reduced nonlinear softening. In partially layered profiles the largest amplification, approximately 5.67, occurred when a thin clay layer overlies thick sand in Profile 06, while the lowest amplification, between about 1.36 and 1.88, occurred in profiles with thick sand at the surface such as Profile 04. Deamplification zones were also identified, varying across profiles and shaking levels. These observations highlight the critical importance of accurately characterizing soil stratigraphy for reliable site-specific seismic hazard assessment and earthquake resistant design.

## Introduction

One of the primary goals of geotechnical engineering is to accurately characterize the soil layers that form the foundation of a structure and to evaluate their behavior under potential earthquake loading before the design and implementation of any engineering project. Understanding the mechanical and dynamic properties of these soil layers is essential because they govern how seismic energy is transmitted, modified, or amplified as it travels from bedrock to the ground surface. Failure to adequately account for these effects can result in unsafe structural designs, underestimated seismic demands, and ultimately, catastrophic damage during earthquakes. For this reason, site-specific ground response analysis has become a fundamental component of seismic hazard assessment, as it allows engineers to incorporate local geotechnical conditions into seismic design frameworks.

Historical earthquake investigations have consistently demonstrated that local geology and soil stratigraphy can significantly alter ground motion characteristics and damage patterns^1,2^. Local site conditions significantly influence the intensity, frequency content, and duration of ground motions during earthquakes. As seismic waves propagate from bedrock to the surface, they interact with overlying soil layers, often undergoing amplification or attenuation depending on the stratigraphic and geotechnical characteristics of the site^3^. This phenomenon, known as site amplification, is a well-established concept in seismology and plays a critical role in determining both the extent of structural damage and its spatial distribution during earthquakes^4,5^. Which is particularly pronounced in regions where soft sediments overlay stiffer bedrock, leading to substantial variations in seismic response across short distances^6–8^.

The direct correlation between local ground conditions and the severity and pattern of building damage has been repeatedly documented in post-earthquake reconnaissance efforts. For instance, the devastating impact of the 1999 Quindío earthquake on Armenia city was closely linked to ground conditions, with damaged buildings heavily concentrated in areas of soft soils and where unplanned urban sprawl occurred in hill and soft-soil areas. Furthermore, in the heavily affected parts of the city, there was a good correlation between filled areas and disaster damage^9^. Research focusing on the 2016 Central Italy earthquakes in Amatrice also highlighted that the extreme damage and high number of collapses were primarily due to the high vulnerability of the old masonry buildings, however, it was noted that the recorded strong motion showed spectral accelerations far exceeding the Italian code, and local site amplification effects related to the town’s location on an alluvial terrace were also a significant concern, illustrating the combined role of structural vulnerability and amplified ground motion^10^.

Beyond single-event observations, studies aiming to quantify seismic risk and loss also underscore the critical dependence on accurate site characterization. For example, a case study in the Luchon Valley, France, investigated the impact of soil characterization maps and their spatial resolution on seismic damage and loss estimates. This study concluded that using less refined, large-scale European soil maps led to a significant underestimation of the proportion of heavily damaged buildings, demonstrating that the resolution of the soil data directly affects loss predictions^11^.

This necessity for integrating local effects into risk assessment has led to the proposal of advanced parameters, such as the HSM (a synthetic damage-constrained seismic hazard parameter). HSM parameter combines the regional seismic hazard with the site amplification factor derived from microzonation studies to create an absolute ranking of seismic hazard in chosen vibration period ranges, directly facilitating the calculation of expected average damage (damage-constrained hazard) and helping decision-makers prioritize mitigation policies^12^. Most recently, comprehensive research performed across the entire Italian territory investigated the influence of local site effects on seismic risk maps and the ranking of municipalities. This work confirmed that introducing new geo-lithological site amplification factors (AFs), which had an average increase of 75% in PGA hazard, led to a significant increase in expected losses compared to models that assumed only rock conditions. This stark increase in predicted losses emphasizes the practical importance of incorporating detailed site-specific amplification into contemporary seismic risk assessments and planning^13^.

Among the most influential parameters governing site amplification are the stratification and composition of the soil column. Vertically heterogeneous profiles, comprising alternating layers of sand, clay, silt, and gravel, introduce complex impedance contrasts that affect wave reflection, refraction, and transmission. These contrasts can result in resonance effects, trapping seismic energy within certain layers and amplifying ground motion at specific frequencies. The precise configuration and succession of lithotypes (soil layers) profoundly influence the seismic response, studies often leverage stochastic or permutation approaches to generate and analyze all possible lithotype successions when subsurface conditions are highly uncertain^14^. The dynamic properties of soils, particularly shear wave velocity (Vs), damping ratio, and nonlinear stress strain behavior, further complicate the seismic response^15–18^.

The fundamental analysis of site amplification is typically conducted using one dimensional model, which assumes that the soil is horizontally layered and that seismic waves propagate vertically through these layers. While one dimensional ground response analysis forms the foundation of seismic site characterization, real geological settings often exhibit more complex geometries. Two dimensional (2D) and three dimensional (3D) effects, such as topographic irregularities, basin edges, and lateral impedance contrasts, can significantly modify seismic motion through wave diffraction, focusing, and interference^19–22^. These effects, commonly referred to as basin or topographic amplification, highlight the importance of advanced numerical modeling in regions with complex subsurface structures. Nevertheless, one dimensional analysis continues to play a central role in site specific studies, providing the fundamental understanding of vertical wave propagation and forming the basis for calibrating and validating higher dimensional models.

A clear understanding of site amplification and its role in shaping earthquake-induced damage is of paramount importance within seismology and earthquake engineering. Over the years, researchers have made substantial progress in investigating this phenomenon using a combination of approaches, such as in situ field monitoring, laboratory testing, and advanced numerical simulations. These methods have provided valuable insights into how local soil conditions and stratigraphic variations contribute to the modification of seismic waves, thereby influencing structural performance during seismic events^23^.

Among these studies, Chala and Ray (2024)^3^ employed one dimensional equivalent linear site response analysis to investigate the effects of local soil profiles on seismic response, demonstrating that variations in stratigraphy and soil composition significantly influence amplification patterns and ground motion characteristics. Likitlersuang et al. (2020)^24^ examined the influence of spatial variability in ground conditions on seismic response and reported that soft soil deposits amplify ground motion during seismic wave propagation. Gobbi et al. (2020)^25^ investigated the impact of variability in soil profile properties under both weak and strong seismic motions, showing that differences in soil layering and material characteristics can result in substantial changes in ground motion behavior. Similarly, Tajabadipour (2022)^26^ analyzed the effects of regional geology and soil conditions using multiple earthquake scenarios with varying peak ground accelerations, finding that increased soil cohesiveness broadens the frequency band and enhances resonance potential, while nonlinear analysis provides a more realistic representation of soil behavior than equivalent-linear methods. Roy et al. (2020)^27^ studied the effects of trapped soft and stiff soil layers on equivalent-linear seismic site response. In addition, Qodri et al. (2021)^28^ reported significant amplification in the soil deposits of Bangkok, with seismic ground motions amplified by factors ranging from approximately 1.41 to 3.45.

Overall, the literature demonstrates that local site conditions, particularly soil stratification, nonlinearity, and impedance contrasts are decisive in shaping ground motion characteristics. For the purpose seismic ground response analysis is generally performed to study the local site effects. Thus, ground response analysis (GRA) has become an integral part in a site-specific seismic hazard study^6,16,27,29–36^.

The influence of soil stratification, particularly the configuration of sand and clay layers, on nonlinear ground response has not yet been systematically evaluated. Although previous studies have examined site amplification in a general sense, the specific effects of varying layer thicknesses and stratigraphic sequences of sand and clay on seismic ground motion amplification remain insufficiently understood. Accordingly, this study aims to comprehensively quantify the influence of different stratified soil configurations, including homogeneous and mixed sand and clay profiles, on nonlinear ground response and ground motion amplification. The role of the upper soil layer, whether sand or clay, and its relative thickness in controlling amplification patterns has also been examined, along with the influence of input ground motions applied at consistent intensity levels to enable a robust comparison across soil profiles. The outcomes of the ground response analysis are presented in terms of peak ground acceleration, response spectra, and amplification factors, providing a detailed understanding of amplification mechanisms in layered soils. Finally, the study investigates the extent to which stratigraphic variability alters seismic wave propagation, spectral shifts, and resonance behavior, offering valuable insights for site specific seismic hazard assessment and earthquake-resistant design.

## Research methodology

**Fig. 1:**
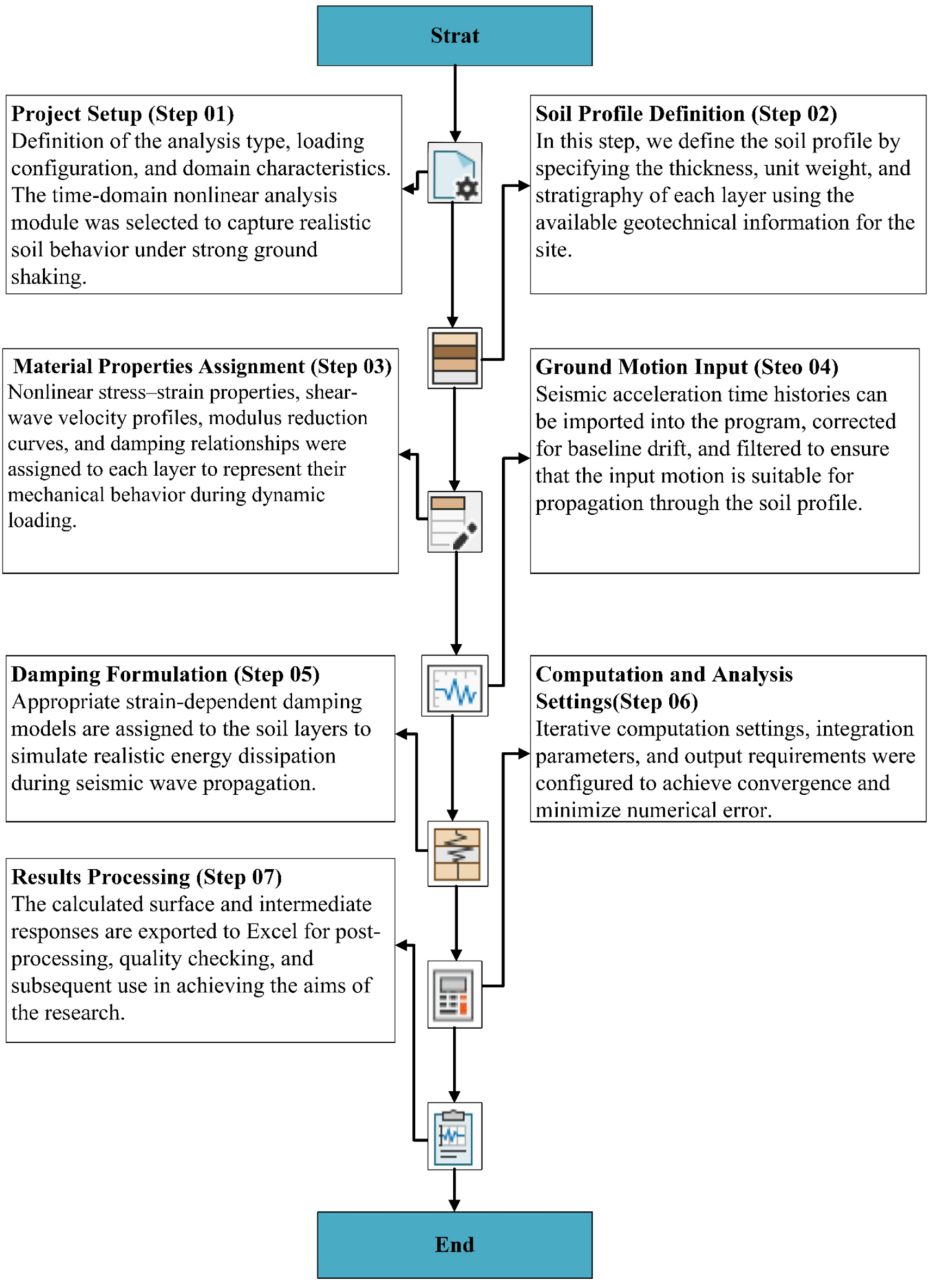
Workflow of the RSSeismic software illustrating the major steps in the ground response analysis.

This study aims to evaluate the influence of different soil types and layer configurations on nonlinear ground response and seismic wave amplification using one dimensional (1D) nonlinear ground response analysis. The research investigates how varying combinations of sand and clay layers affect peak ground acceleration (PGA), surface spectral acceleration (Sa), and amplification factors (AF). Numerical simulations were performed using RSSeismic v.1 software, released on July 31, 2025, by Rocscience Inc^37^, under an exclusive license from the university of Illinois Urbana Champaign. RSSeismic represents the continuation of the widely used DEEPSOIL platform^38^, maintaining full compatibility with DEEPSOIL files while introducing enhanced capabilities for seismic ground response analysis. The software is a one dimensional ground response analysis program that simulates the propagation of seismic waves through horizontally layered soil deposits. It allows equivalent linear analyses in both the time and frequency domains, nonlinear analyses in the time domain, and fully linear analyses in both domains. In this study, the nonlinear time domain approach was adopted to capture soil stiffness degradation and damping behavior under strong ground motion. RSSeismic employs a finite difference formulation in the time domain to solve the 1D wave equation for vertically propagating shear waves. The program provides several constitutive models for simulating strain dependent soil behavior and computes acceleration, velocity, and displacement responses at user defined depths. The complete workflow of the RSSeismic software used in this study is summarized and illustrated in Fig. 1, outlining all major steps involved in performing 1D ground response analysis from project setup and soil profile definition to damping formulation, computation, and results processing.

A total of eight stratigraphic models (profiles) were developed to examine the influence of soil layering. All profiles had a total depth of 30 m and varied in the thickness and sequence of clay and sand layers, which will be discussed in detail in the following section. Each layer was assigned soil-specific properties such as unit weight, shear wave velocity, modulus reduction, and damping ratio. Nonlinear soil behavior was primarily modeled using the General Quadratic/Hyperbolic (GQ/H) model, which was selected as the default constitutive model in the software. The GQ/H model is a generalized hyperbolic formulation that defines soil strength and stiffness degradation under cyclic loading. To accurately capture the cyclic hysteretic behavior, the non-masing unloading and reloading rule was applied as the default hysteretic formulation. This rule is an advanced modification within the GQ/H framework that corrects the traditional masing behavior by introducing a reduction factor during cyclic reloading. As a result, the model can simultaneously reproduce both the target modulus reduction (G/Gmax) and damping ratio curves, ensuring more realistic representation of energy dissipation and stiffness degradation in nonlinear time-domain analyses^39^.

**Fig. 2:**
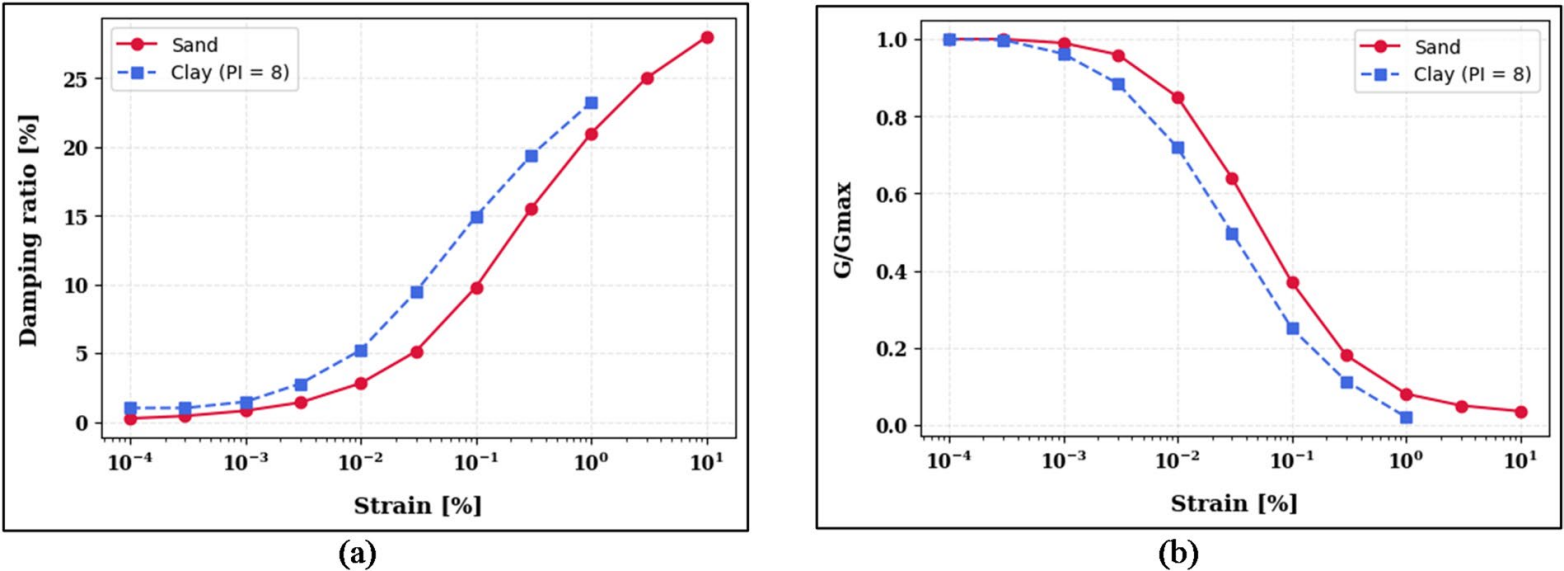
The panel (**a**) presents the normalized shear modulus reduction curves (G/Gmax) for sand and clay (PI = 8). The panel (**b**) shows the corresponding damping ratio curves for both soils.

Modulus reduction and damping curves were adopted from Seed and Idriss (1970, upper limit) for sand^40^, and from Vucetic and Dobry (1991) for clay^41^. According to the site geotechnical investigation report, the groundwater level was not encountered up to a depth of 30 m in the borehole, and the clay’s plasticity index was determined as 8.2%, which falls within the low-plasticity range (PI < 15%). Therefore, the corresponding Vucetic and Dobry (1991) relationships appropriate for low-plasticity clay were applied in the analysis. These curves account for strain-dependent nonlinearity and hysteretic damping (see Fig. 2). Table 1 presents the key material properties used in the ground response analysis, while Fig. 2 illustrates the corresponding modulus reduction and damping ratio curves for sand and clay.

**Fig. 3:**
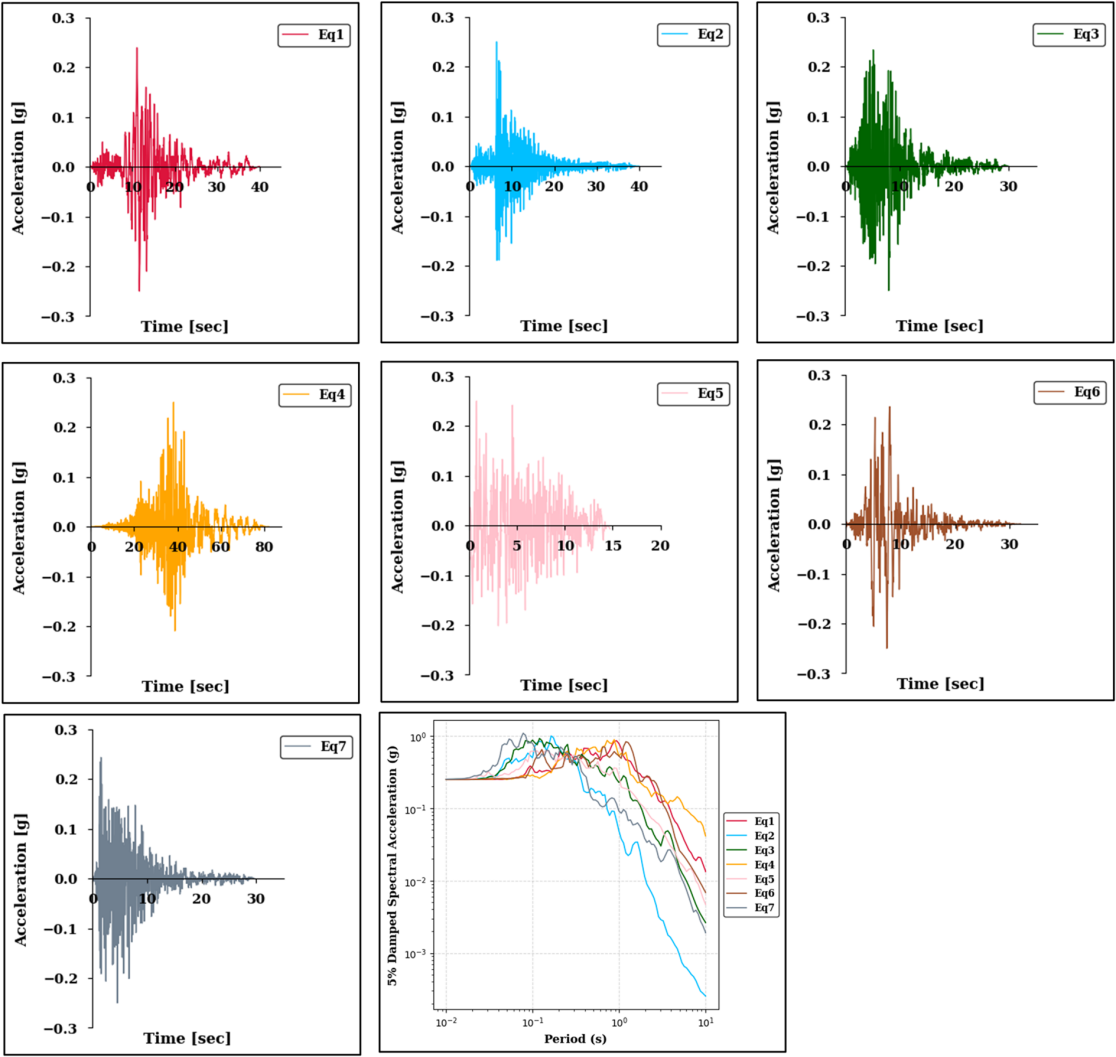
Time histories (Eq. 1-Eq. 7) and 5% damped spectral acceleration curves of input ground motions scaled to 0.25 g. Only the 0.25 g case is presented for illustration, as similar trends were observed across other intensity levels (0.10 g and 0.50 g).

Seven strong ground motion records were selected from the Pacific Earthquake Engineering Research Center (PEER) Updated NGA WEST2 Flatfile Vertical 5% Damping Dataset^42^ using SeismoSignal version 4.3 software^43^. These motions were scaled to 0.10 g, 0.25 g, and 0.50 g to represent low, moderate, and high intensity input motions, respectively. The applied scale factors ranged from approximately 0.36 to 6.55 for the 0.10 g level, from 0.91 to 16.39 for the 0.25 g level, and from 1.81 to 32.77 for the 0.50 g level. All records represent rock motions (soil class A) with characteristics suitable for nonlinear analysis and were applied uniformly at the base of each soil column at a depth of 30 m. Table 2 presents the key parameters of the input ground motion records used in the analysis, while Fig. 3 presents the time histories and corresponding 5% damped spectral acceleration curves of the input ground motions scaled to 0.25 g. Although three intensity levels (0.10 g, 0.25 g, and 0.50 g) were considered in the analysis, only the 0.25 g case is shown here for visual clarity.

In this study, 1D free field boundary conditions were applied to simulate vertically propagating shear waves in a horizontally layered soil column. Because the analysis is one dimensional, the vertical sides of the model are assumed to behave identically, and there is no lateral strain or shear stress transfer between adjacent columns, representing a condition of infinite horizontal extent. The base of the model was treated as a rigid bedrock boundary, where the input acceleration time histories were applied as vertically propagating shear waves. Analyses were conducted in the time domain using the non-masing unloading and reloading rule to better reflect cyclic loading behavior and energy dissipation^44^.

Ground response was assessed in terms of peak ground acceleration at the surface, 5% damp response spectra, and the amplification factor, defined as the ratio between the spectral acceleration responses at the soil surface and those corresponding to the input motion at the reference bedrock (see Eq. 1), assessed across the entire period range of interest^45^. Comparative analyses were performed across all stratification scenarios to evaluate how the order and thickness of sand and clay layers influenced nonlinear amplification behavior. Finally, the results and conclusions are presented accordingly.

1 $$\:AF=\frac{{PGA}_{surface}}{{PGA}_{input\:motion}}$$

**Table 1:** Key material properties used in the ground response analysis. “Top” and “Bottom” denote the upper and lower boundaries of each soil layer, respectively.

Identification	Soil type	Layer thickness (m)	Shear wave velocity (m/s)		Unit weight (kN/m^3^)
			Top	Bottom	
Profile 01	Clay/sand	15/15	180/360	320/620	16/18
Profile 02	Sand/clay	15/15	360/180	620/320	18/16
Profile 03	Sand/clay	7.5/22.5	360/180	620/320	18/16
Profile 04	Sand/clay	22.5/7.5	360/180	620/320	18/16
Profile 05	Sand	30	360	620	18
Profile 06	Clay/sand	7.5/22.5	180/360	320/620	16/18
Profile 07	Clay/sand	22.5/7.5	180/360	320/620	16/18
Profile 08	Clay	30	180/360	320/620	16

### Nonlinear ground response analysis (GRA)

Nonlinear GRA is a time-domain seismic analysis approach used to evaluate the dynamic behavior of soil deposits subjected to earthquake loading, explicitly accounting for the nonlinear stress–strain response of soils at varying shear strain levels. Unlike linear or equivalent linear methods, which assume constant or iteratively adjusted soil properties, nonlinear GRA directly captures strain-dependent variations in soil stiffness and damping during seismic excitation, thereby providing a more realistic representation of soil behavior under strong ground motions.

The soil column is discretized using either a finite element formulation or a multi-degree-of-freedom lumped-parameter model, and the governing dynamic equilibrium equation is formulated in the time domain^44,46^. The equation of motion for the soil system is expressed in matrix form as:

2 $$\:\left[M\right]\left\{\ddot{u}\right\}+\:\left[C\right]\left\{\dot{u}\right\}+\:\left[K\right]\left\{u\right\}=\:-\left[M\right]\left\{I\right\}{\ddot{u}}_{g}$$

where $\:\left[M\right]$, $\:\left[C\right]$, and $\:\left[K\right]$denote the mass, viscous damping, and stiffness matrices of the soil profile, respectively; $\:\left\{u\right\}$, $\:\left\{\dot{u}\right\}$, and $\:\left\{\ddot{u}\right\}$represent the nodal relative displacement, velocity, and acceleration vectors of the soil layers; $\:\left\{I\right\}$ is a unit vector; and $\:{\ddot{u}}_{g}$is the input ground acceleration applied at the base of the soil column. The dynamic equilibrium equation (Eq. 2) is solved incrementally at each time step using the Newmark-β time integration scheme^47^. At each time increment, the soil stiffness and damping properties are updated iteratively based on the computed shear strain levels, ensuring that the nonlinear response of the soil is accurately represented throughout the duration of seismic loading.

**Table 2:** Key parameters of input ground motion records used in the seismic analysis.

ID	Earthquake name	Year	Station name	Mw	Fault type	Rrup (km)	5–95% duration (s)	Vs30 (m/s)
Earthquake 1	Loma Prieta	1989	SF - Pacific Heights	6.93	Reverse Oblique	76.05	11.90	1249.86
Earthquake 2	Whittier Narrows-01	1987	Vasquez Rocks Park	5.99	Reverse Oblique	50.39	9.20	996.43
Earthquake 3	Northridge-01	1994	LA - Wonderland Ave	6.69	Reverse	20.29	8.70	1222.52
Earthquake 4	Chi-Chi_ Taiwan	1999	ILA015	7.62	Reverse Oblique	85.40	28.00	782.59
Earthquake 5	San Fernando	1971	Cedar Springs Allen Ranch	6.61	Reverse	89.72	10.40	813.48
Earthquake 6	Kobe_ Japan	1995	Kobe University	6.90	strike slip	0.92	7.00	1043.00
Earthquake 7	Morgan Hill	1984	Gilroy Array #1	6.19	strike slip	14.91	9.50	1428.14

### Site profile development

Eight distinct synthetic soil profiles were developed to simulate a variety of soil configurations commonly encountered in geotechnical and seismic site investigations. Each profile was modeled to a depth of 30 m and consists of sand and/or clay layers in varying proportions and sequences. These configurations were designed to investigate the influence of soil type, layer thickness, and position (upper or lower) on seismic ground response.

The profiles are as follows:

- Profile 01 (ClSa 50:50): A 15 m clay layer overlying a 15 m sand layer, representing an equal division of both soils with clay in the upper half.
- Profile 02 (SaCl 50:50): A 15 m sand layer overlying a 15 m clay layer, also a 50–50 configuration but with sand on top.
- Profile 03 (SaCl 25:75): A 7.5 m sand layer overlying 22.5 m of clay, representing a profile with 25% sand and 75% clay (sand on top).
- Profile 04 (SaCl 75:25): A 22.5 m sand layer overlying 7.5 m of clay, corresponding to 75% sand and 25% clay with sand on top.
- Profile 05 (Sa100): A homogeneous profile consisting of 30 m of sand overlying bedrock.
- Profile 06 (ClSa 25:75): A 7.5 m clay layer overlying 22.5 m of sand, representing 25% clay at the top and 75% sand below.
- Profile 07 (ClSa 75:25): A 22.5 m clay layer overlying 7.5 m of sand, representing 75% clay and 25% sand (clay on top).
- Profile 08 (Cl100): A homogeneous profile consisting of 30 m of clay overlying bedrock.

These profiles capture a wide range of soil layering scenarios to allow evaluation of both uniform and mixed conditions. The upper layer type and thickness were of particular interest, as they are most responsible for modifying the amplitude and frequency characteristics of seismic waves.

Each profile was modeled with a consistent total thickness (30 m) to ensure direct comparison of response behavior, while the material properties (e.g., density, shear wave velocity, damping) were assigned based on the respective soil type (clay or sand). The soil profiles are automatically discretized into sublayers to ensure accurate wave propagation modeling. This process begins after defining the maximum propagating frequency, typically set to around 30 Hz, which represents the upper limit containing approximately 95% of the total energy of the input ground motions. Using the specified maximum frequency, the software computes the highest frequency that each soil layer can transmit based on its shear wave velocity and thickness, defined as f_max_=Vs/4H, where Vs is the shear wave velocity and H is the layer thickness. Each layer is then automatically divided into sublayers of equal thickness to satisfy the required frequency resolution. The properties of these sublayers remain the same as those of the parent layer, ensuring consistent stiffness and damping characteristics throughout the soil column. This automatic discretization approach enhances the numerical accuracy of wave propagation within the one-dimensional ground response model and ensures that all significant frequencies of engineering interest are properly captured in the analysis^39^.

The frequency content of the input ground motions plays a crucial role in controlling seismic resonance and the dynamic response of soil profiles. In this study, the profiles natural periods ($\:{T}_{0}$) range from 0.25 s to 0.49 s, while the input motions exhibit predominant periods ($\:{T}_{p}$) between 0.08 s and 1.22 s. Under low-intensity conditions, resonance occurs when $\:{T}_{p}\approx\:{T}_{0}$, producing maximum amplification. At higher motion intensities, nonlinear soil behavior reduces stiffness and elongates the effective natural period, shifting the amplification peak toward longer periods.

**Fig. 4:**
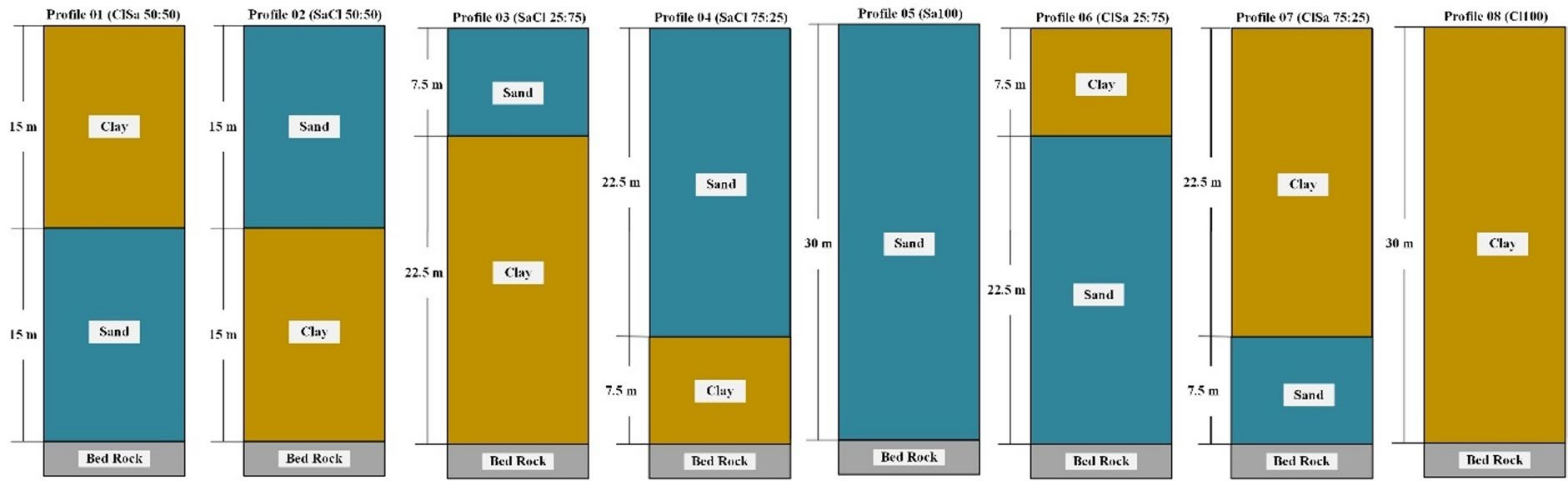
Schematic illustration of the eight soil profiles used in the study. Each profile consists of a combination of clay and sand layers with varying thicknesses over a 30 m soil column.

Figure 4 shows the schematic configuration of the eight site profiles used in this study, categorized based on the vertical distribution of clay and sand layers within the upper 30 m of soil. The profiles vary in terms of the thickness and position of sand and clay layers, allowing for a comparative analysis of their effects on ground motion amplification.

## Results and discussion

This section presents the results of the nonlinear ground response analyses performed for all soil profiles. To provide a clear and systematic evaluation, the eight profiles are first organized into four groups according to the proportion and vertical arrangement of sand and clay. Within each group, the relevant soil profiles are introduced first, followed by three subsections that present the analysis results for the corresponding input ground motion levels of 0.10 g, 0.25 g, and 0.50 g. A comprehensive interpretation of the amplification patterns, de-amplification ranges, and nonlinear soil response characteristics is then provided in the final discussion section.

### 100% homogeneous soil profiles

#### Analysis for 0.10 g PGA

**Fig. 5:**
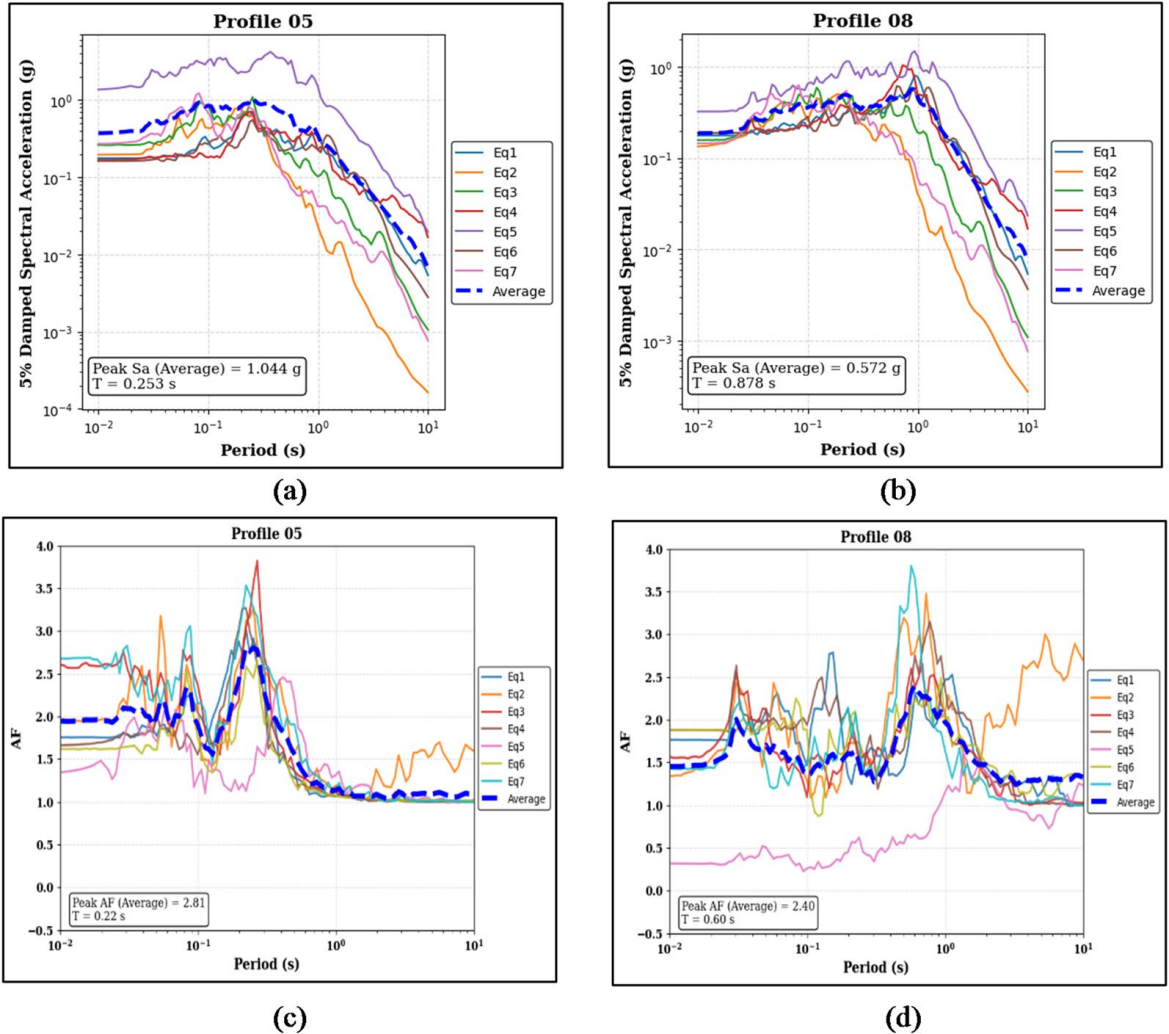
Panels (**a**) and (**b**) show the 5% damped Sa curves and median peak Sa values for the sand-over-bedrock (Profile 05) and clay-over-bedrock (Profile 08) profiles, respectively. Panels (**c**) and (**d**) present the corresponding AF curves and median peak AF values for the same profiles.

Figure 5a and b present the 5% damped spectral acceleration (Sa) results for the two 30 m homogeneous soil profiles. For the sand-over-bedrock profile (Profile 05), the median peak Sa is 1.044 g at a period of 0.253 s, while for the clay-over-bedrock profile (Profile 08), the median peak Sa is 0.572 g at a period of 0.878 s. The corresponding amplification factor (AF) results are shown in Fig. 5c and d. Profile 05 exhibits a median peak AF of 2.81 at a period of 0.22 s, whereas Profile 08 shows a median peak AF of 2.40 at a period of 0.60 s. These values represent the peak amplification trends for each profile under the applied ground motion records.

#### Analysis for 0.25 g PGA

**Fig. 6:**
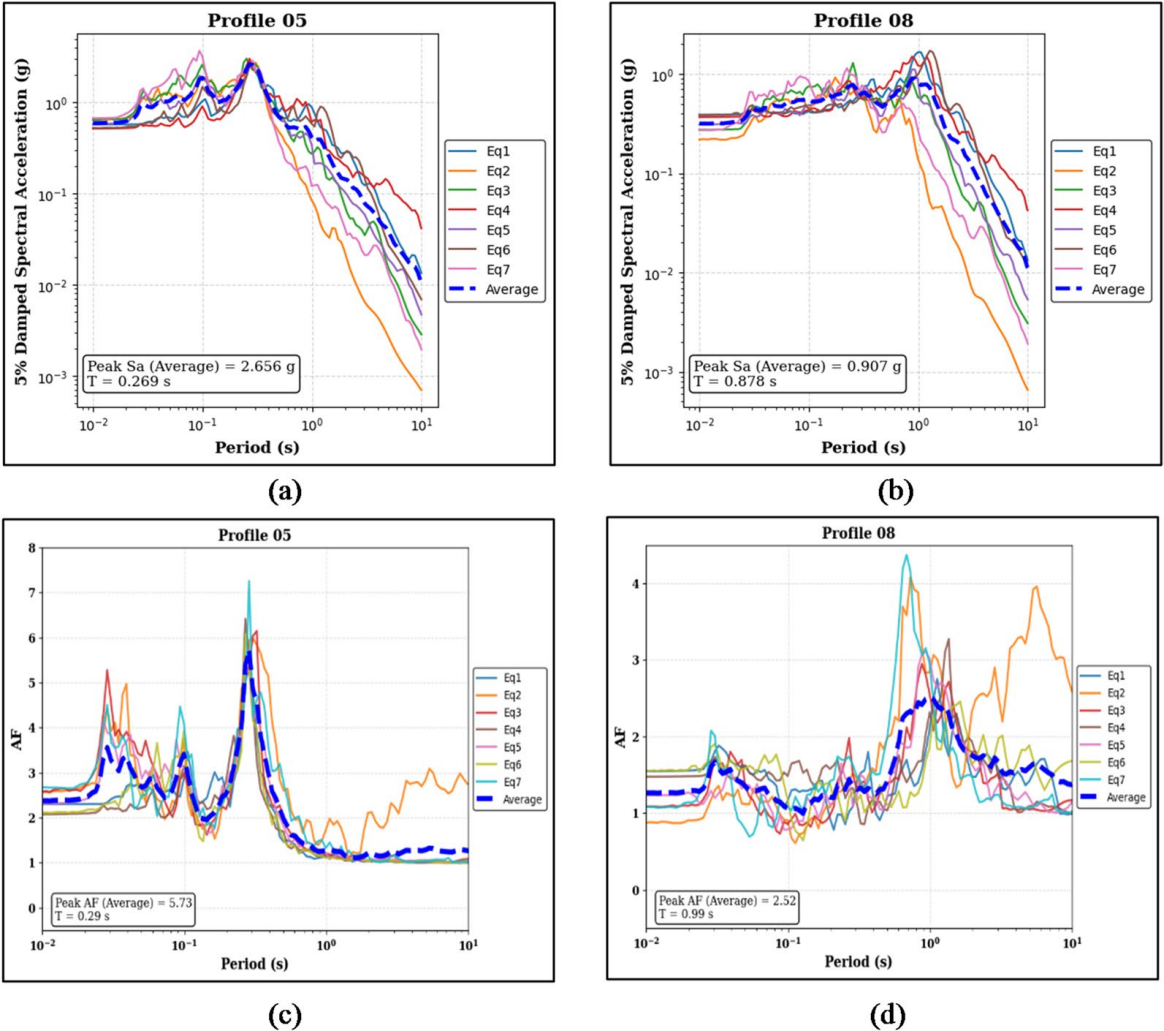
Panels (**a**) and (**b**) show the 5% damped Sa curves and median peak Sa values for the sand-over-bedrock (Profile 05) and clay-over-bedrock (Profile 08) profiles, respectively. Panels (**c**) and (**d**) present the corresponding AF curves and median peak AF values for the same profiles

The analysis results for 0.25 g PGA conducted on 100% homogeneous soil profiles are presented here, the composition and layer sequences are same as discussed for the 0.10 g PGA case (see Sect. 3.1.1). Figure 6a and b show the median peak Sa is 2.656 g for Profile 05 at a T of 0.269 s, and 0.907 g for Profile 08 at a T of 0.878 s.

Figure 6c,d present the corresponding AF results. Profile 05 exhibits a median peak AF of 5.73 at a period of 0.29 s, whereas Profile 08 shows a median peak AF of 2.52 at a period of 0.99 s.

#### Analysis for 0.50 g PGA

**Fig. 7:**
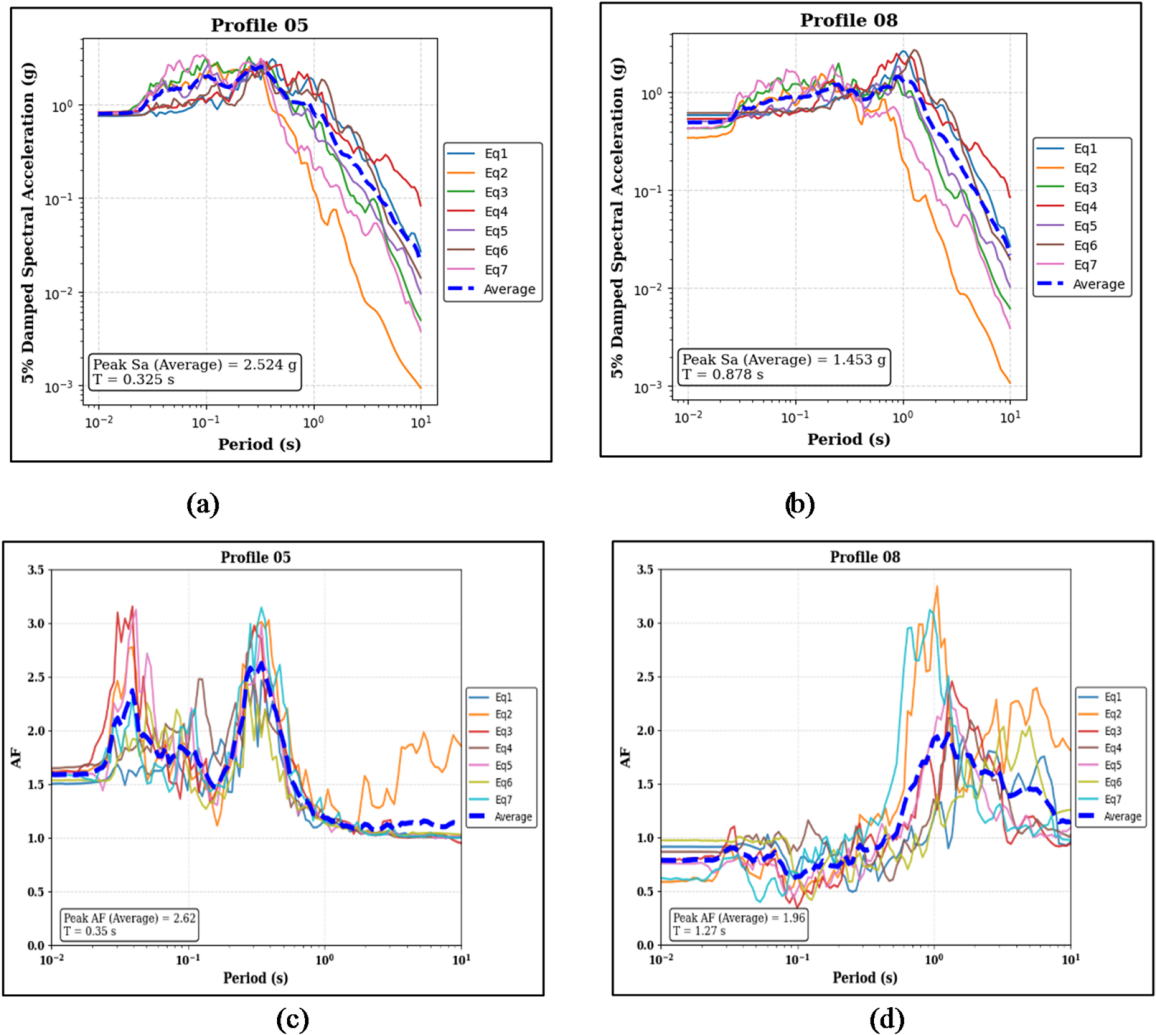
Panels (**a**) and (**b**) show the 5% damped Sa curves and median peak Sa values for the sand-over-bedrock (Profile 05) and clay-over-bedrock (Profile 08) profiles, respectively. Panels (**c**) and (**d**) present the corresponding AF curves and median peak AF values for the same profiles

Figure 7 shows the analysis results of the 100% homogeneous profiles as discussed in the previous sections. The soil configuration and layering sequence are the same as discussed in Sect. 3.1.1, while the analysis is performed for a PGA of 0.50 g.

Figure 7a,b illustrate that the median Sa for Profile 05 is 2.524 g at a T of 0.325 s, and 1.453 g at a T of 0.878 s for Profile 08. The corresponding median peak AF is 2.62 at a period of 0.35 s for Profile 05 (Fig. 7c), and 1.96 at a period of 1.27 s for Profile 08 (Fig. 7d).

### 75%–25% layered soil profiles

In addition to the fully homogeneous soil profiles, analyses were also performed for partially layered soil profiles to investigate the effect of stratification on seismic site response. In this section, two configurations were considered: profile 07, consisting of 22.5 m of clay underlain by 7.5 m of sand (clay on top), and profile 04, consisting of 22.5 m of sand overlying 7.5 m of clay (sand on top). These profiles represent stratified deposits with 75%–25% distributions of clay and sand in different layering sequences. The results of the analysis for different PGA levels are included in the blow subsections.

#### Analysis for 0.10 g PGA

**Fig. 8:**
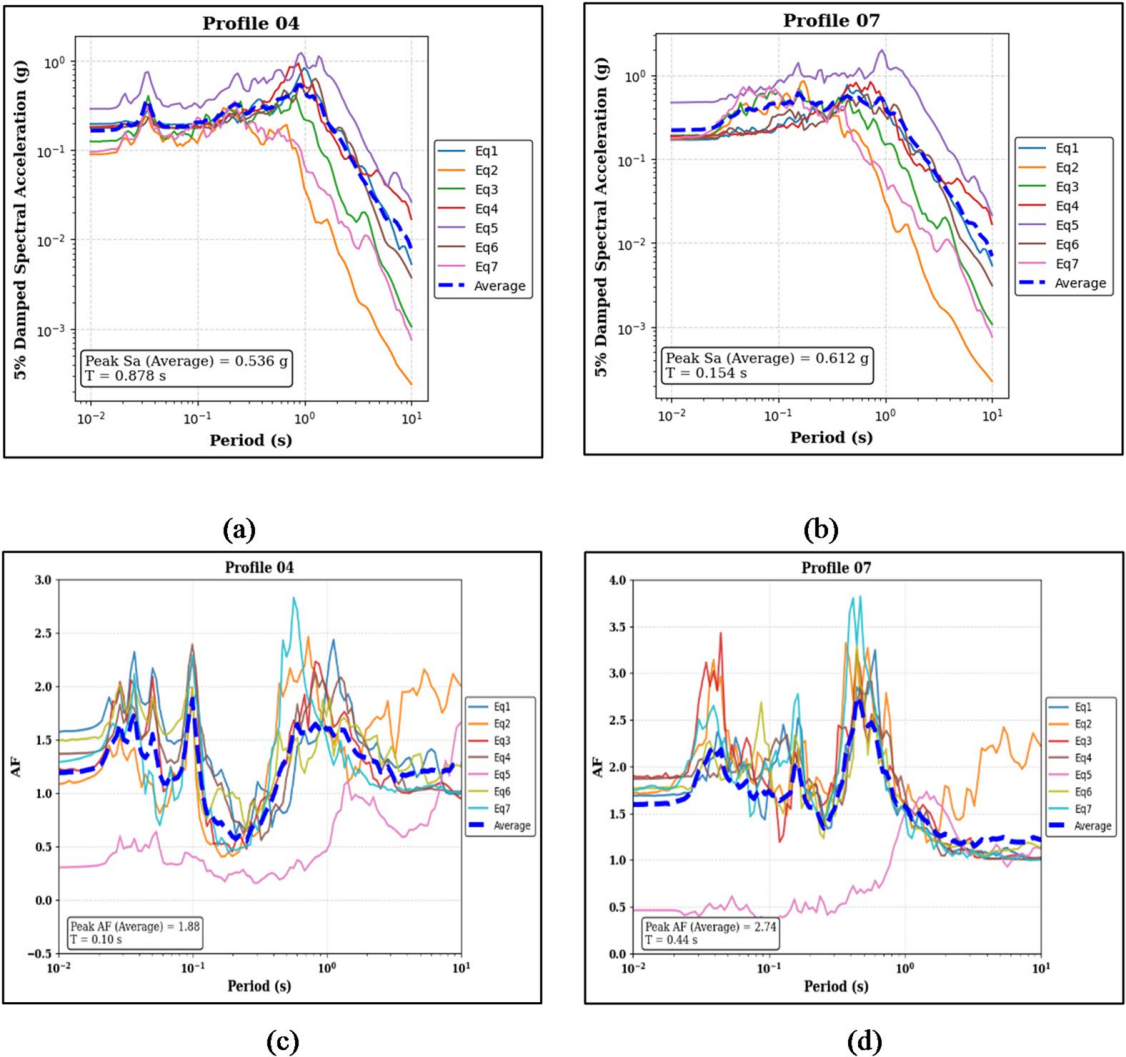
Panels (**a**) and (**b**) show the 5% damped Sa curves and median peak Sa values for the sand-on-top (Profile 04) and clay-on-top (Profile 07) profiles, respectively. Panels (**c**) and (**d**) present the corresponding AF curves and median peak AF values for the same profiles.

Figure 8a, b present the 5% damped spectral acceleration (Sa) results for the two 30 m partially layered soil profiles. For the sand-on-top profile (Profile 04), the median peak Sa is 0.536 g at a period of 0.878 s, whereas for the clay-on-top profile (Profile 07), the median peak Sa reaches 0.612 g at a period of 0.154 s. Figure 8c and d explicitly illustrate the corresponding AF results for Profiles 04 and 07, respectively. Profile 04 exhibits a median peak AF of 1.88 at a period of 0.10 s, while Profile 07 shows a median peak AF of 2.74 at a period of 0.44 s.

#### Analysis for 0.25 g PGA

**Fig. 9:**
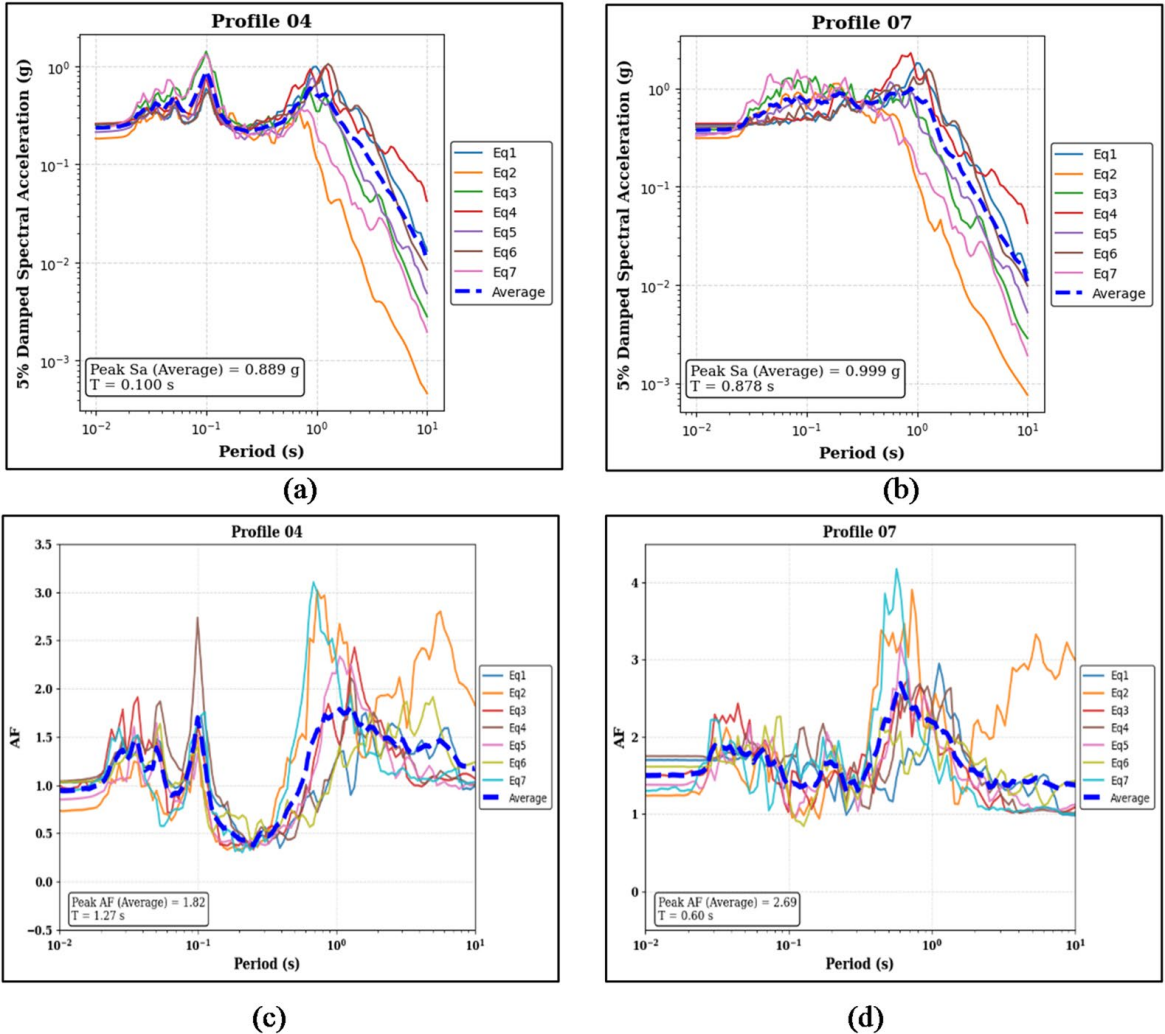
Panels (**a**) and (**b**) show the 5% damped Sa curves and median peak Sa values for the sand-on-top (Profile 04) and clay-on-top (Profile 07) profiles, respectively. Panels (**c**) and (**d**) present the corresponding AF curves and median peak AF values for the same profiles.

Figure 9a, b present the results for the partially layered profiles. For Profile 04, the median peak Sa is 0.889 g at a period of 0.100 s, while for Profile 07, the median peak Sa is 0.999 g at a period of 0.878 s.

The corresponding amplification factor results are shown in Fig. 9c and d. Profile 04 exhibits a median peak AF of 1.82 at a period of 1.27 s, whereas Profile 07 shows a median peak AF of 2.69 at a period of 0.60 s.

#### Analysis for 0.50 g PGA

**Fig. 10:**
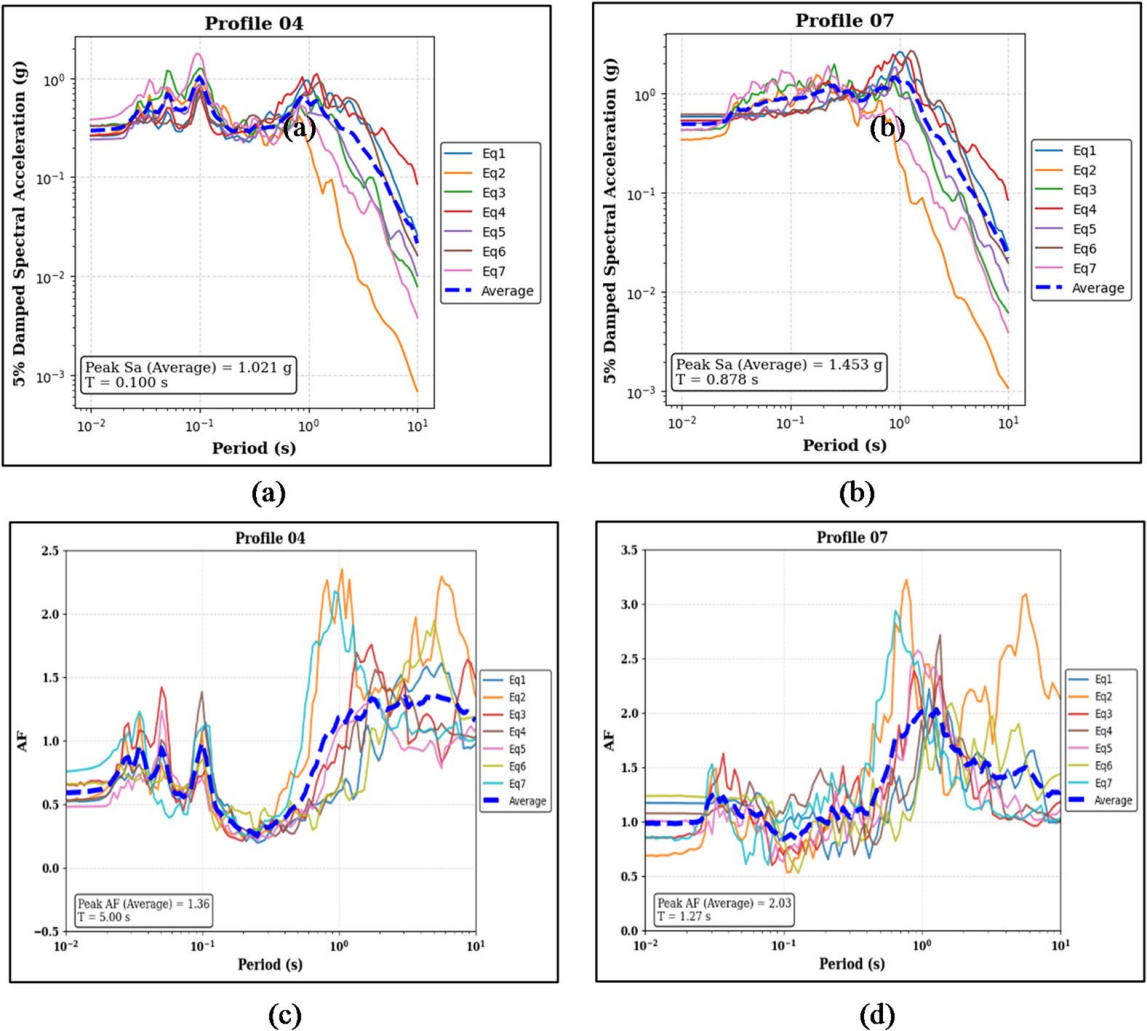
Panels (**a**) and (**b**) show the 5% damped Sa curves and median peak Sa values for the sand-on-top (Profile 04) and clay-on-top (Profile 07) profiles, respectively. Panels (**c**) and (**d**) present the corresponding AF curves and median peak AF values for the same profiles.

Figure 10a, b delineate the median peak Sa is 1.021 g at T of 0.100 s (Profile 04). In contrast, Profile 07 exhibits a median peak Sa of 1.453 g at a longer period of 0.878 s. The corresponding AF metrics are featured in Fig. 10c and d. Profile 04 manifests a median peak AF of 1.36 at a period of 0.80 s, whereas Profile 07 records a median peak AF of 2.03 at a period of 1.27 s.

### 25%–75% layered soil profiles

In this section Profile 03 comprises a 7.5 m sand layer overlying 22.5 m of clay, illustrating a configuration with sand at the surface and clay below. Profile 06 consists of a 7.5 m clay layer overlying 22.5 m of sand, representing a soil column with clay at the surface and sand beneath.

#### Analysis for 0.10 g PGA

**Fig. 11:**
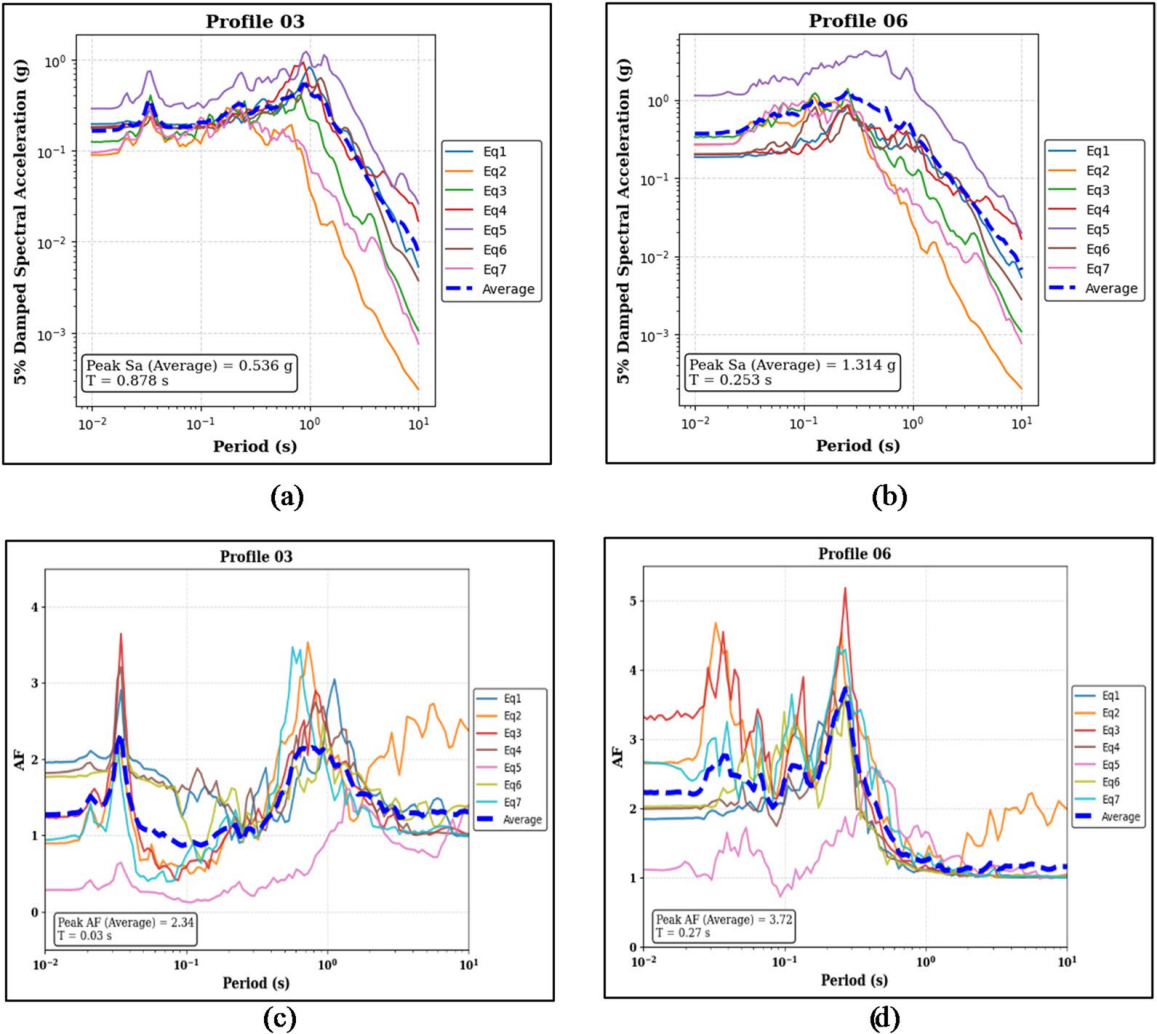
Panels (**a**) and (**b**) show the 5% damped Sa curves and median peak Sa values for the sand-on-top (Profile 03) and clay-on-top (Profile 06) profiles, respectively. Panels (**c**) and (**d**) present the corresponding AF curves and median peak AF values for the same profiles.

Figure 11a and b present the 5% damped Sa results for the two 30 m partially layered soil profiles. For Profile 03, the median peak Sa is 0.536 g at a period of 0.878 s, while for Profile 06, the median peak Sa is 1.314 g at a period of 0.253 s. The associated AF spectra are presented in Fig. 11c and d. Profile 03 records a median peak AF of 2.34 at a short period of 0.03 s, while Profile 06 exhibits a higher median peak AF of 3.72 at a period of 0.27 s.

#### Analysis for 0.25 g PGA

**Fig. 12:**
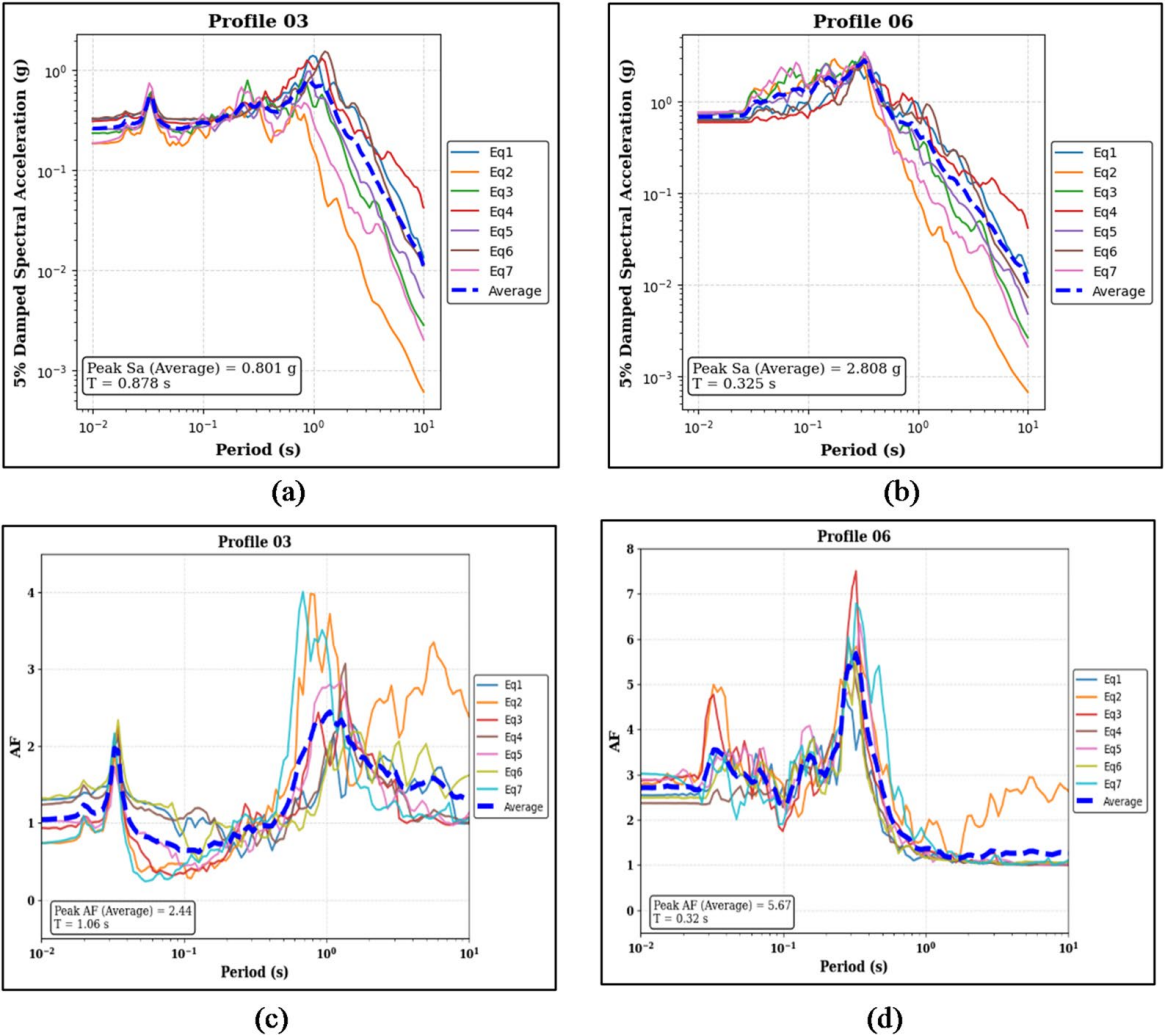
Panels (**a**) and (**b**) show the 5% damped Sa curves and median peak Sa values for the sand-on-top (Profile 03) and clay-on-top (Profile 06) profiles, respectively. Panels (**c**) and (**d**) present the corresponding AF curves and median peak AF values for the same profiles.

Figure 12a and b present the 5% damped Sa results for the two profiles. For Profile 03, the median peak Sa is 0.801 g at a period of 0.878 s, while Profile 06 shows a higher median peak Sa of 2.808 g at a shorter period of 0.325 s.

Figure 12c,d show the corresponding AF results. Profile 03 exhibits a median peak AF of 2.44 at a period of 1.06 s, whereas Profile 06 records a higher median peak AF of 5.67 at a period of 0.32 s

#### Analysis for 0.50 g PGA

**Fig. 13:**
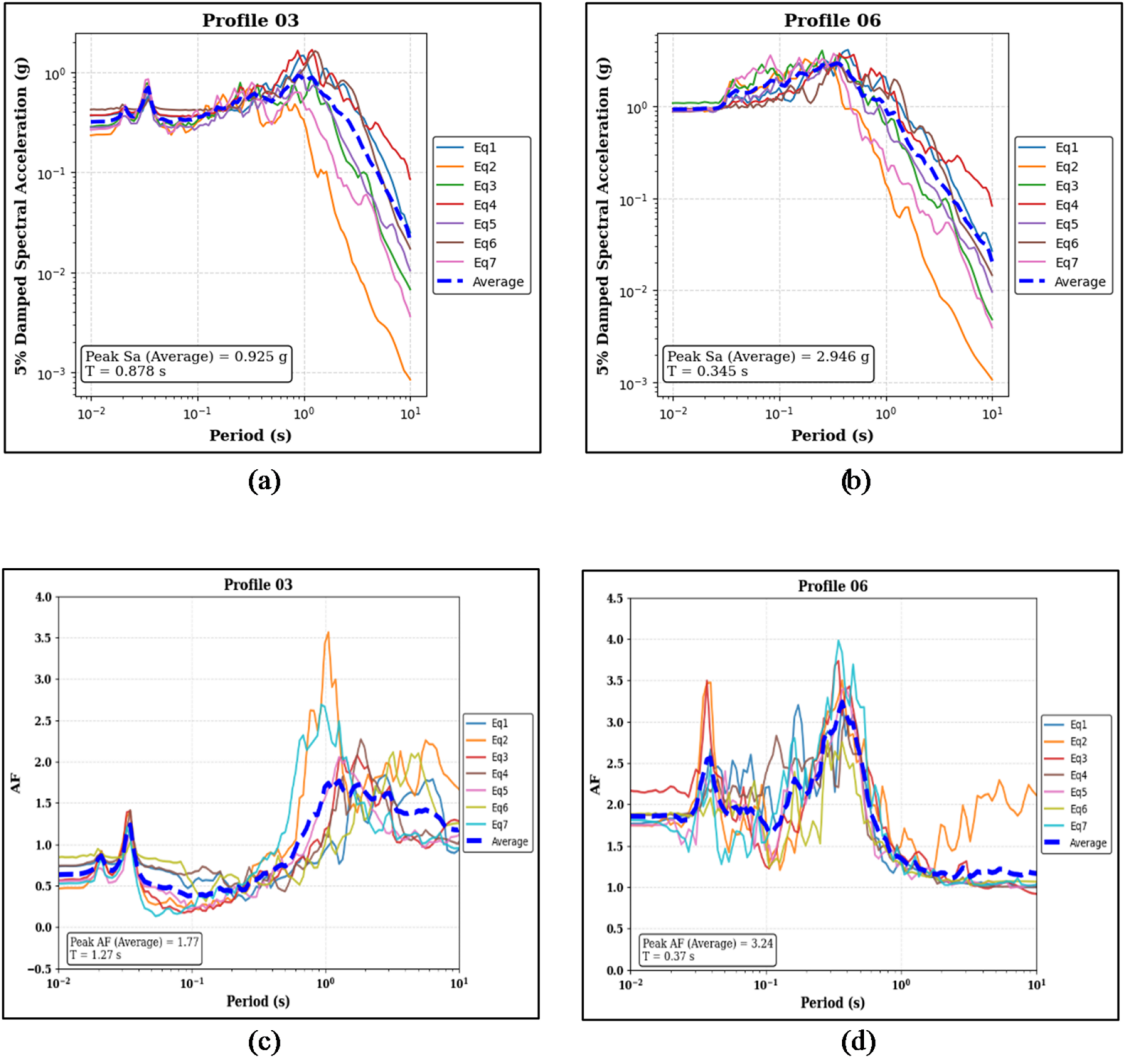
Panels (**a**) and (**b**) show the 5% damped Sa curves and median peak Sa values for the sand-on-top (Profile 03) and clay-on-top (Profile 06) profiles, respectively. Panels (**c**) and (**d**) present the corresponding AF curves and median peak AF values for the same profiles.

Figure 13a, b appear to indicate differing spectral acceleration behaviors for the two profiles. For Profile 03, the Sa curve suggests a peak value around 0.925 g occurring at a longer period of approximately 0.878 s. Profile 06, on the other hand, seems to show a noticeably higher peak Sa, approaching 2.946 g, which is associated with a shorter period near 0.343 s.

A similar pattern can be observed in the amplification factor results shown in Fig. 13c and d. Profile 03 appears to reach a median peak AF of about 1.77 at a period close to 1.27 s. Profile 06 seems to develop an amplification response, with its peak AF of roughly 3.24 occurring at a shorter period of around 0.37 s.

### 50%–50% layered soil profiles

In this part of the study, the focus shifts to two 30 m deep soil profiles characterized by an equal distribution of clay and sand. Profile 01 features a 15 m clay layer resting atop 15 m of sand, whereas Profile 02 has a reversed arrangement, with 15 m of sand overlying 15 m of clay. These configurations allow for a comparative assessment of how the position of each soil type affects the seismic response of a half-and-half stratified profile.

#### Analysis for 0.10 g PGA

**Fig. 14:**
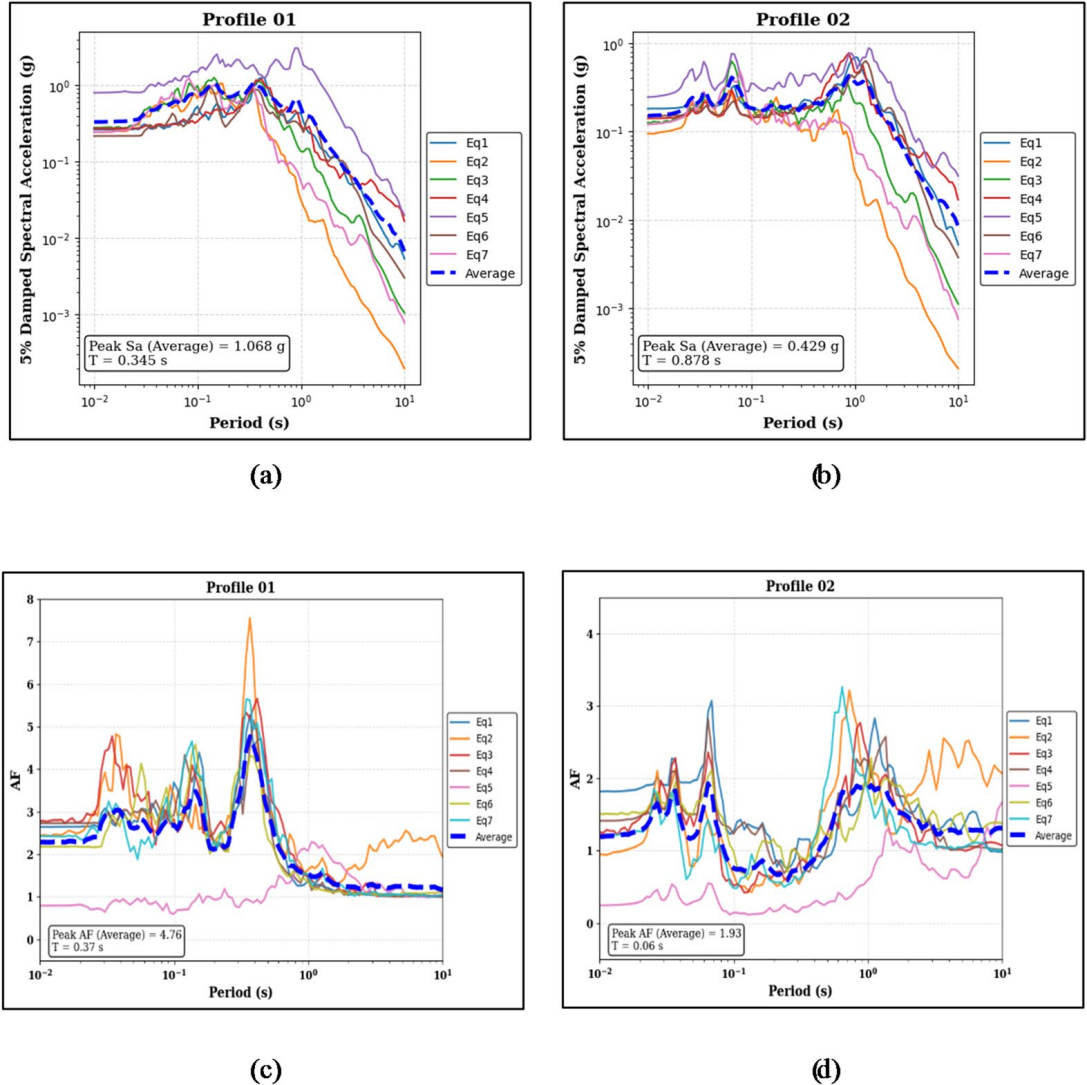
Panels (**a**) and (**b**) show the 5% damped Sa curves and median peak Sa values for the clay-on-top (Profile 01) and sand-on-top (Profile 02) profiles, respectively. Panels (**c**) and (**d**) present the corresponding AF curves and median peak AF values for the same profiles.

The computed results, displayed in Fig. 14, demonstrate that the arrangement of clay and sand significantly impacts both peak spectral acceleration and amplification behavior. Profile 01, with clay at the surface, shows median peak Sa of 1.068 g at a period of 0.345 s, while the sand-capped Profile 02 exhibits 0.429 g at 0.878 s (see Fig. 14a and b). Amplification trends further emphasize the role of surface material, with profile 01 reaching a median AF of 4.76 at 0.370 s, compared to 1.93 at 0.060 s for profile 02 (see Fig. 14c and d).

#### Analysis for 0.25 g PGA

**Fig. 15:**
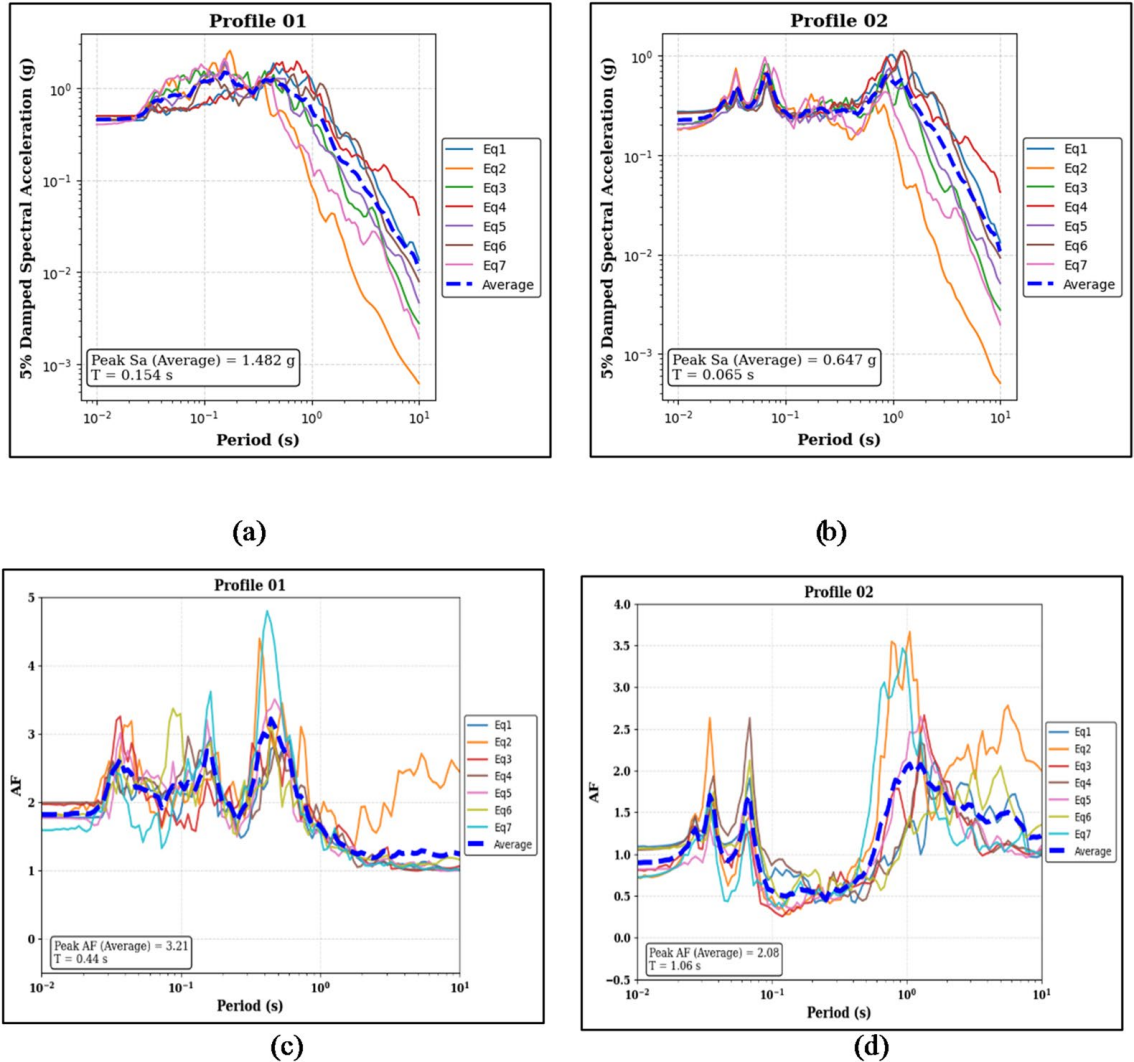
Panels (**a**) and (**b**) show the 5% damped Sa curves and median peak Sa values for the clay-on-top (Profile 01) and sand-on-top (Profile 02) profiles, respectively. Panels (**c**) and (**d**) present the corresponding AF curves and median peak AF values for the same profiles.

The computed results, displayed in Figs. 15, demonstrate that the arrangement of clay and sand significantly impacts both peak spectral acceleration and amplification behavior. Profile 01, with clay at the surface, shows median Sa of 1.482 g at a period of 0.154 s, while the sand-capped Profile 02 exhibits 0.647 g at 0.065 s (see Fig. 15a and b). Amplification trends further emphasize the role of surface material, with Profile 01 reaching a median amplification factor of 3.21 at 0.440 s, compared to 2.08 at 1.0.6 s for Profile 02 (see Fig. 15c and d).

#### Analysis for 0.50 g PGA

**Fig. 16:**
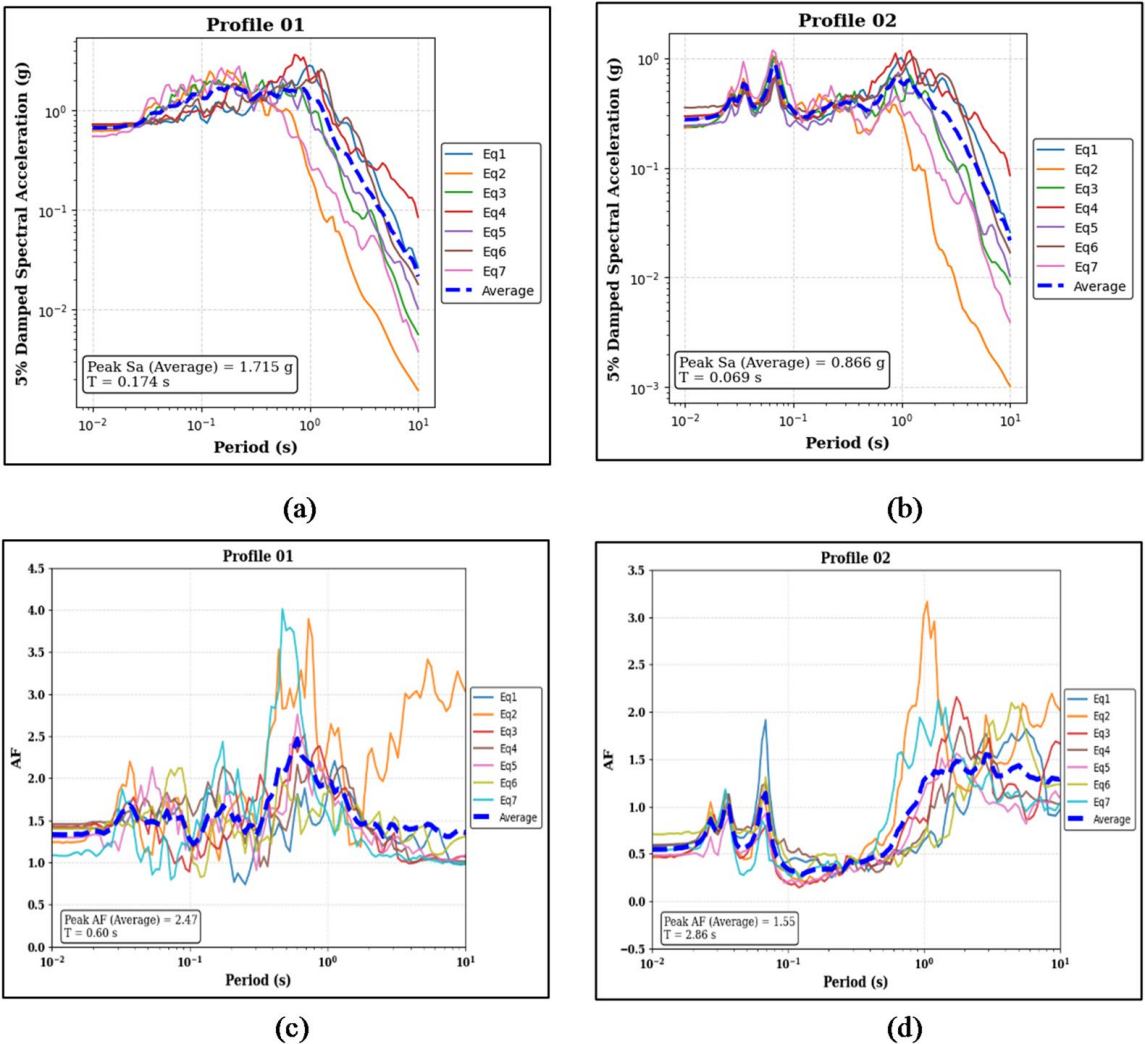
Panels (**a**) and (**b**) show the 5% damped Sa curves and median peak Sa values for the clay-on-top (Profile 01) and sand-on-top (Profile 02) profiles, respectively. Panels (**c**) and (**d**) present the corresponding AF curves and median peak AF values for the same profiles.

Figure 16a and b present the 5% damped Sa results for Profile 01 and Profile 02. Profile 01 shows a median peak Sa of 1.715 g at a T of 0.174 s, whereas Profile 02 exhibits a lower median peak Sa of 0.866 g at a very short period of 0.069 s. Figure 16c and d show the corresponding AF results. Profile 01 records a median peak AF of 2.47 at a period of 0.60 s, while Profile 02 demonstrates a median peak AF of 1.55 at a longer period of 2.86 s.

### Discussion

The nonlinear ground response analyses clearly show that ground motion amplification in stratified soil profiles is strongly controlled by soil type, impedance contrast, and the vertical arrangement of clay and sand layers. For all input PGA levels, a consistent pattern is observed: sand layers, with higher stiffness and shear-wave velocity, tend to amplify short-period ground motions, while clay layers shift amplification toward longer periods due to their lower stiffness and greater nonlinear degradation. The behavior of the uppermost soil layer is especially influential because it governs the impedance mismatch with the underlying materials and controls the amount of nonlinear softening during strong shaking.

**1. Amplification and de-amplification characteristics**.

Amplification factors across the eight profiles vary substantially, with AF values generally falling between about 1.36 and 5.73 depending on PGA and soil stratigraphy.

Large amplification tends to occur when a thin clay layer overlies a thick sand layer or when strong impedance contrast exists between adjacent layers. For example, Profile 06 (7.5 m clay over 22.5 m sand) produced an AF of about 5.67 at a period near 0.32 s under a 0.25 g input motion. This behavior reflects both resonance within the softer upper layer and the stiffness contrast with the material below.

Lower amplification is seen in profiles with thick sand at the surface, since the high stiffness of sand lowers the natural period of the soil column and limits nonlinear strain development. Profile 04 (22.5 m sand over 7.5 m clay) consistently produced smaller AF values, typically between 1.36 and 1.88 across all input motions.

De-amplification in the average response curves was observed for several soil profiles, although within different period ranges. The reported period ranges are based on visual inspection of the plotted response curves and therefore represent approximate values. For Profile 02, de-amplification generally occurred between approximately 0.08 and 0.80 s for PGA levels of 0.50 g and 0.25 g. In Profile 03, the reduction was mainly observed within the period range of 0.50 to 0.70 s for both PGA levels. Profile 04 exhibited de-amplification over a broader period band, approximately from 0.10 to 0.80 s, whereas Profile 07 showed a much narrower range, approximately between 0.10 and 0.20 s. In the homogeneous sand Profile 05, no de-amplification was observed. Finally, the homogeneous clay Profile 08 exhibited de-amplification predominantly within the period range of 0.01 to 0.80 s.

**2. Effect of soil sequence (layer ordering)**.

The vertical ordering of clay and sand layers has a greater effect on ground motion amplification than simply the total amount of each material. Across all input motion levels, profiles with clay at the surface consistently produce higher amplification than those with sand at the surface.

For example, Profile 01 (clay over sand) generated an AF of approximately 3.21 under a 0.25 g input motion, whereas Profile 02 (sand over clay) produced an AF of only about 2.08 at a different period. Similarly, Profile 07 (22.5 m clay over 7.5 m sand) shows stronger amplification at intermediate periods compared to Profile 04, even though both profiles contain the same total layers thicknesses.

**3. Influence of layer thickness**.

The thickness of individual soil layers determines the natural period of the soil column and therefore shifts the periods at which amplification occurs.

Thick clay layers, such as the 22.5 m layer in Profile 07, result in amplification at longer periods near about 0.6–1.27 s. Thinner clay layers, for example in Profile 06 (7.5 m), cause amplification at shorter or intermediate periods such as 0.27–0.37 s. Thick sand layers shorten the natural period of the soil column and suppress long-period amplification.

As examples, Profile 07 with a thick clay layer produced an AF of about 2.69 at approximately 0.6 s, while Profile 06 with a thin clay layer produced a much larger AF of about 5.67 at a shorter period of roughly 0.32 s. This difference is due to the stronger nonlinear softening induced in thinner, soft layers subjected to larger shear strain demands.

**4. Role of Nonlinear Soil Behavior**.

As the level of shaking increases from 0.10 g to 0.25 g to 0.50 g, all profiles exhibit measurable nonlinear effects, including period elongation and shifting of amplification peaks. Clay-rich profiles show the most significant period lengthening because they experience more shear modulus degradation.

In sand layers, increasing strain causes greater material damping, which reduces high-frequency amplification. For example, in Profile 05, the AF at 0.50 g becomes smaller than at 0.25 g, indicating that strong-motion damping is suppressing amplification.

These trends are consistent with established nonlinear site response theory and demonstrate that increasing seismic intensity alters the frequency content and magnitude of site amplification, particularly in profiles where soft layers are present near the ground surface.

## Conclusion

This study systematically evaluated the nonlinear seismic response of eight stratified soil profiles to quantify the effects of soil type, layer thickness, and vertical ordering on ground motion amplification. The results demonstrate that site response is not governed solely by the presence of clay or sand but is predominantly influenced by the material located near the ground surface, the impedance contrast between layers, and the degree of nonlinear degradation developed under different shaking intensities.

Across all three input ground motion levels of 0.10 g, 0.25 g, and 0.50 g, the strongest amplification consistently occurred in profiles containing thin clay layers overlying thicker sand layers. These configurations produced large impedance contrasts and pronounced nonlinear softening, resulting in amplification factors of up to approximately 5.67 in Profile 06 under an input motion of 0.25 g. In contrast, profiles with thick sand layers at the surface, such as Profile 04, exhibited the lowest amplification, with amplification factors ranging from approximately 1.36 to 1.88. This behavior is attributed to the lower nonlinear strain demand and shorter natural periods associated with stiffer sand layers.

The comparison of layer ordering revealed that stratigraphic sequence is more influential than the total proportion of each soil type. Although Profiles 01 and 02 contain equal volumes of sand and clay, Profile 01, with clay at the surface, consistently produced higher amplification than Profile 02, where sand forms the upper layer. This finding indicates that stratigraphic arrangement directly controls resonance behavior and the periods at which amplification peaks occur.

Layer thickness was also shown to affect the dominant amplification periods. Thick clay layers tended to shift amplification toward longer periods, approximately between 0.6 and 1.27 s, while thin clay layers produced stronger amplification at shorter to intermediate periods. These results highlight the importance of accurately identifying layer thickness during geotechnical investigations, particularly when performing nonlinear site response analyses.

Nonlinear soil behavior became increasingly significant with increasing shaking intensity. Period elongation, increased damping, and reduced amplification were observed under stronger input motions, particularly in stiff soil profiles. Clay-rich profiles exhibited the most pronounced nonlinear effects, leading to larger shifts in spectral peaks and broader amplification regions.

The study has the following limitations. The analyses were conducted using a 1D vertical wave propagation model assuming horizontally layered soil conditions and therefore do not account for 2D or 3D effects. Only two-layer soil profiles were considered, whereas natural soil deposits often consist of multiple layers with more complex stratigraphic arrangements. In addition, shear-wave velocity profiles and modulus reduction and damping curves were assumed to be fixed and representative of each soil type, while natural variability in soil properties may result in a wider range of responses at real sites. These assumptions define the scope of the analysis and should be considered when interpreting the results.

The principal contribution of this study is the demonstration that soil layer ordering and thickness exert a greater control on nonlinear ground motion amplification than the overall proportion of sand and clay within a soil profile. Based on these findings, it is recommended that site-specific seismic assessments place particular emphasis on accurately identifying near-surface soil conditions and layer thickness rather than relying solely on averaged soil properties. Future studies may extend this work by considering multi-layer soil profiles and incorporating two dimensional or three dimensional site effects.

## Data Availability

The necessary data used in the manuscript is already present in the manuscript.

## Declarations

### Transparency statement

The authors affirm that this manuscript provides a truthful, accurate, and transparent account of the research. No significant aspects of the study have been omitted, and any deviations from the original study plan (and, if applicable, registration) have been fully explained.

### Declaration of AI use

The authors declare not use AI-assisted technologies in creating this article.
